# The Association of High Prevalence of Trophozoites in Peripheral Blood with Lower Antibody Response to* P. falciparum* Infected Erythrocytes among Asymptomatic Children in Sudan

**DOI:** 10.1155/2016/7987686

**Published:** 2016-06-28

**Authors:** Sara N. Mohamed, Dina A. Hassan, Abdelrahim M. El Hussein, Ihssan M. Osman, Muntasir E. Ibrahim, Hiba S. Mohamed, Bakri Y. Nour, Nasreldin H. Abdulhadi

**Affiliations:** ^1^Central Laboratory, Ministry of Higher Education and Scientific Research, Khartoum, Sudan; ^2^Faculty of Medicine, Al-Zaiem Al Azhari University, Khartoum, Sudan; ^3^Institute of Endemic Diseases, University of Khartoum, 11111 Khartoum, Sudan; ^4^The Blue Nile Institute for Training and Research, Gezira University, Wad Madani, Sudan; ^5^College of Pharmacy, The National Ribat University, Khartoum, Sudan

## Abstract

*Background*. The most prominent variant surface antigens (VSAs) of* Plasmodium falciparum* are the var gene-encoded* Plasmodium falciparum* erythrocyte membrane protein 1 (PfEMP1) family, which serves as a parasite-sequestering ligand to endothelial cells. In this study we have examined the antibody reactivity of autologous plasma from symptomatic and asymptomatic malaria infected children against the infected erythrocytes' surface antigens using flow cytometry.* Methods*. Ethidium-bromide-labelled erythrocytic mature forms of* P. falciparum* parasites obtained from symptomatic and asymptomatic children were sequentially incubated with autologous plasma and fluorescein isothiocyanate-conjugated (FITC) antihuman IgG. Plasma antibody reactivity was detected by flow cytometry.* Results*. Asymptomatic children had more prevalence of trophozoites in peripheral blood (66%) compared to symptomatic children (16%), *p* = 0.002. The mean percentage of infected RBCs reacting with autologous sera was 89.78 among symptomatic children compared to 79.62 among asymptomatic children (*p* = 0.09). Moreover, the mean fluorescence intensity (MFI) in the asymptomatic was significantly higher compared to symptomatic children (*p* value = 0.040).* Conclusion*. Variant surface antigens on* Plasmodium falciparum* infected RBCs from symptomatic malaria children tend to be better recognized by IgG antibodies. This may suggest a role of some IgG antibodies in severity of malaria.

## 1. Background

The variant surface antigens (VSAs) including the most recognized* Plasmodium falciparum* erythrocyte membrane protein 1 (PfEMP1) are responsible for the pathogenicity of malaria infection. Through interaction with host molecules such as ICAM1, CD36, CR1, and CD31, VSAs play a central role in mediating cytoadherence of infected erythrocytes to host cells. This is believed to be responsible for the severe pathology associated with* P. falciparum* malaria [1]. Parasite-infected erythrocytes from young children and those with severe malaria were shown to be better recognized by antibodies from semi-immune children than those from older children or those with nonsevere malaria [2, 3]. Levels of antibodies to many malaria antigens may vary with the seasonality of malaria transmission, often being higher during periods of high malaria transmission than at the end of a low transmission season [4–8]. Antibodies to malaria antigens often tend to be higher in individuals who are infected at the time their antibodies are measured than in noninfected individuals [2, 9, 10]. The predominance of IgG1 and IgG3 cytophilic antibodies in endemic areas has been associated with either lower parasitaemia [11] or a lower risk of malaria attack [12, 13]. Noncytophilic antibodies, such as IgG4, may inhibit effector mechanisms by competing with cytophilic antibodies and are considered nonprotective [14, 15]. IgG2 is noncytophilic but could be correlated with protection in individuals carrying a specific allelic variant of monocytes Fc*γ*RIIA receptor that can bind IgG2 [16]. Most antibody studies use laboratory strains as source of antigens which may not reflect antigen repertoire of field strains. This preliminary study is intended to measure total plasma IgG antibody response to VSA antigens using autologous plasma among symptomatic and asymptomatic children. The levels of antibodies so determined were then correlated to the reduced iRBCs cytoadherence phenomenon, previously reported by our group as a possible determinant of asymptomatic malaria among Fallata tribe children in Sudan [17].

## 2. Subjects and Methods

The study was approved by the Ethical Committee of the Institute of Endemic Diseases, University of Khartoum, Sudan. It was conducted in Al Keraiba, a suburb of Wad Madani, central Sudan, during November-December 2009, at the peak of malaria transmission in this area. A total of 55 children, belonging to the Fallata tribe, of whom 24 presented with fever with or without other symptoms of malaria and 31 were asymptomatic, are included in this study.* P. falciparum* infection was confirmed by ICT test and microscopy. Three mL venous blood sample was obtained in citrated vacutainer tube from each child after obtaining signed consent forms from their parents. The symptomatic children were obtained from Al Keraiba health center while the asymptomatic children were obtained during a cross-sectional survey for malaria in Al Keraiba primary school. Asymptomatic children were followed up for 48 hours and 8 of them who developed symptoms within the follow-up period were excluded.

### 2.1. Parasitaemia

Levels of initial parasitaemia (parasites/*μ*L) were determined by counting parasites against 200 leukocytes in the thick smears taken at the time of sampling assuming that each subject had 8000 leukocytes/*μ*L.

### 2.2. Parasite Maturation and Fixation

Infected erythrocytes were obtained from each sample, washed in RPMI medium, and grown in culture for 48–60 hours [18]. Trophozoites/schizonts so obtained were enriched by gel floatation [18] and preserved in transfix solution (Transfix® Cytomark Ltd., Buckingham, England).

### 2.3. Flow Cytometry

Trophozoite/schizont infected RBCs (iRBCs) obtained after gel floatation and fixation were incubated with 1 : 200 diluted autologous plasma in FACS staining buffer (FSB) for 45 minutes at room temperature. Samples were washed twice with FSB and incubated with fluorescein-conjugated antihuman IgG (Sigma, UK) and 2 *μ*g/mL ethidium bromide at room temperature for 30 minutes. Samples were then washed twice with FBS. Coulter EPICS XL-MCL flow cytometer was used for acquirement and analysis of 10000 events/sample. Populations of parasitized erythrocytes expressing antiserum-reactive VSA (EtdBr- and plasma antibodies-positive) were gated. The mean fluorescence intensities (MFI) were determined for symptomatic and asymptomatic groups.

### 2.4. Statistics

Statistical significance was calculated by two-sample, two-tailed Student's* t*-test (SPSS Software, version 13.0). Comparisons were considered statistically significant at *p* < 0.05.

## 3. Results

There were no significant differences between symptomatic and asymptomatic children in terms of age; however the mean starting parasitaemia was significantly higher among symptomatic children (4.59 ± 0.52 parasite/*μ*L) compared to asymptomatic children (1.33 ± 0.52 parasite/*μ*L). All symptomatic and asymptomatic samples gave positive antibody response after counting for the background given by noninfected RBCs. The mean (±SEM) percentage of infected RBCs reacting with autologous sera (VSA-positive cells) was 89.78 ± 1.67 among symptomatic children compared to 79.62 ± 2.97 among asymptomatic children (*p* = 0.09). The mean fluorescence intensity, MFI (±SEM), in the symptomatic (32.65 ± 10.3) was significantly higher compared to asymptomatic children (8.2 ± 1.0) (*p* value = 0.04, Table 1).

## 4. Discussion

Previously we have reported an* in vivo* reduced sequestration of infected erythrocytes among asymptomatic children in the suburbs of Wad Madani, the same area of the present study [17]. It is well known that the major antigenic ligands found to be responsible for the cytoadhesive properties of the infected red blood cells (iRBCs) are members of the* P. falciparum* erythrocyte membrane protein 1 (PfEMP1) family. These variable surface antigens (VSAs) are placed on knob-like structures on the surface of the iRBCs and bind to different host vascular adhesins, including CD36, ICAM1, VCAM1, and CSA [19]. We assumed a differential antibody response to the VSA of the iRBCs that might block or mediate cytoadhesion leading to asymptomatic or symptomatic disease, respectively. However, levels of antibodies to many malaria antigens may vary with the seasonality of malaria transmission [7]. Antibodies often tend to be higher in individuals who also have malaria parasites at the time their antibodies are measured than in those without parasites [2]. Moreover, follow-up studies intended to define protective immune responses against malaria are usually handicapped. This is attributable to a difficulty in distinguishing between individuals who do not get clinical episodes because they were unexposed and those who are truly immune. We assume that having the infection at the time of blood collection and using autologous plasma to look at antibody response may overcome the exposure heterogeneity and brevity of antibody response as a measure of immunity [20]. In this study, the lower antibody response among asymptomatic children which was accompanied with reduced cytoadherence, as evident by higher percentage of circulating trophozoites, may suggest that some antibodies can mediate cytoadhesion and lead to immune responses that manifest as clinical presentation. Alternatively, the symptomatic group may also represent cases of very early symptomatic infections in nonimmune children. Our results showed clearly a quantitative difference of total IgG antibody response between symptomatic and asymptomatic children. These results may encourage further detailed qualitative studies of subclasses of antibodies developed during malaria infection to show their possible role in clinical protection in this population.

## 5. Conclusion

The preliminary finding in this study of reduced antibody response to* P. falciparum* malaria among clinically protected children and its association with the reduced cytoadhesion suggests a role for antibodies in sequestration. This hypothesis may be tested by further longitudinal studies to investigate the possible role of different classes and subclasses of immunoglobulins and the effects of age, season, and frequency of infections.

## Figures and Tables

**Table 1:** Age, parasitaemia, prevalence of trophozoites, % VSA, and MFI.

Group	Age (year) M ± SEM	Parasitaemia (parasites/*μ*L)^*∗*^ M ± SEM	Prevalence of trophozoites (%)	% VSA	MFI M ± SEM
Symptomatic (*N* = 24)	8.5 ± 2	4.59 ± 0.52	16	89.78 ± 1.6	32.65 ± 10.3
Asymptomatic (*N* = 23)	8.8 ± 0.6	1.33 ± 0.45	66	79.62 ± 2.9	8.24 ± 1
*p value*	0.990	0.027	0.002	0.090	0.040

^**∗**^×10^3^.
